# Gut Microbiota, Metabolome, and Body Composition Signatures of Response to Therapy in Patients with Advanced Melanoma

**DOI:** 10.3390/ijms241411611

**Published:** 2023-07-18

**Authors:** Giulia Vandoni, Federica D’Amico, Marco Fabbrini, Luigi Mariani, Sabina Sieri, Amanda Casirati, Lorenza Di Guardo, Michele Del Vecchio, Andrea Anichini, Roberta Mortarini, Francesco Sgambelluri, Giuseppe Celano, Nadia Serale, Maria De Angelis, Patrizia Brigidi, Cecilia Gavazzi, Silvia Turroni

**Affiliations:** 1 Clinical Nutrition Unit, Fondazione IRCCS Istituto Nazionale dei Tumori, 20133 Milan, Italy; 2 Microbiomics Unit, Department of Medical and Surgical Sciences, University of Bologna, 40138 Bologna, Italy; 3 Unit of Microbiome Science and Biotechnology, Department of Pharmacy and Biotechnology, University of Bologna, 40126 Bologna, Italy; 4 Data Science Unit, Fondazione IRCCS Istituito Nazionale dei Tumori, 20133 Milan, Italy; 5 Epidemiology and Prevention Unit, Fondazione IRCCS Istituto Nazionale dei Tumori, 20133 Milan, Italy; 6 Clinical Nutrition and Dietetics Unit, Fondazione IRCCS Policlinico San Matteo, 27100 Pavia, Italy; 7 Melanoma Medical Oncology Unit, Fondazione IRCCS Istituto Nazionale dei Tumori, 20133 Milan, Italy; 8 Human Tumors Immunobiology Unit, Department of Research, Fondazione IRCCS Istituto Nazionale dei Tumori, 20133 Milan, Italyroberta.mortarini@istitutotumori.mi.it (R.M.);; 9 Department of Soil, Plant and Food Science (DiSSPA), University of Bari Aldo Moro, 70126 Bari, Italy

**Keywords:** gut microbiota, metabolome, body composition, advanced melanoma, response to therapy

## Abstract

Despite the recent breakthroughs in targeted and immunotherapy for melanoma, the overall survival rate remains low. In recent years, considerable attention has been paid to the gut microbiota and other modifiable patient factors (e.g., diet and body composition), though their role in influencing therapeutic responses has yet to be defined. Here, we characterized a cohort of 31 patients with unresectable IIIC-IV-stage cutaneous melanoma prior to initiation of targeted or first-line immunotherapy via the following methods: (i) fecal microbiome and metabolome via 16S rRNA amplicon sequencing and gas chromatography/mass spectrometry, respectively, and (ii) anthropometry, body composition, nutritional status, physical activity, biochemical parameters, and immunoprofiling. According to our data, patients subsequently classified as responders were obese (i.e., with high body mass index and high levels of total, visceral, subcutaneous, and intramuscular adipose tissue), non-sarcopenic, and enriched in certain fecal taxa (e.g., *Phascolarctobacterium*) and metabolites (e.g., anethole), which were potentially endowed with immunostimulatory and oncoprotective activities. On the other hand, non-response was associated with increased proportions of *Streptococcus*, *Actinomyces*, *Veillonella*, *Dorea*, *Fusobacterium*, higher neutrophil levels (and a higher neutrophil-to-lymphocyte ratio), and higher fecal levels of butyric acid and its esters, which also correlated with decreased survival. This exploratory study provides an integrated list of potential early prognostic biomarkers that could improve the clinical management of patients with advanced melanoma, in particular by guiding the design of adjuvant therapeutic strategies to improve treatment response and support long-term health improvement.

## 1. Introduction

The incidence of melanoma is increasing worldwide, and several studies suggest that that the number of cases has even doubled in the last 10 years (https://www.epicentro.iss.it/melanoma, accessed on 1 September 2022). Fortunately, the advent of targeted therapy and immunotherapy has significantly improved the prognosis, and advanced (unresectable–IIIC and metastatic-IV) melanoma has shifted from being a deadly disease to a disease with effective treatments. However, only a limited subset of patients actually benefit from these treatments, with an overall 3-year survival rate of approximately 50% [1,2].

Based on the recent literature, several modifiable patient-level factors, such as diet, exercise, body composition, and gut microbiota (GM), may influence melanoma progression and therapeutic response [3]. In particular, GM has shown great promise as a biomarker of clinical benefit and a therapeutic target. Indeed, both experimental and human studies have demonstrated the importance of GM in modulating the efficacy of anticancer therapy and patients’ susceptibility to side effects [4]. With particular reference to melanoma, GM is known to play a leading role in the therapeutic response, especially to immune checkpoint inhibitors, by promoting local and systemic inflammation or inducing immunosuppressive phenotypes, thereby enhancing or counteracting the anti-tumor immune response [5,6,7,8,9]. This awareness has paved the way for several clinical trials aimed at manipulating GM (e.g., through diet, bacterial consortia, or even fecal microbiota transplantation) toward a more favorable profile associated with better prognosis and overall survival [10,11,12,13]. However, reliable and consistent GM signatures of response to therapy have yet to be identified [14]. No less important is the fact that insights into the functional contribution of GM (such as those provided via metagenomics, metatranscriptomics, or metabolomics) are still very scarce, although they may provide new and interesting opportunities for adjuvant treatment [13]. Regarding body composition, obesity is an established risk factor for several malignancies, though it was unexpectedly associated with better outcomes in patients with metastatic melanoma who received targeted therapy or immunotherapy [3], thus suggesting a stage- and treatment-dependent relationship. Similarly, sarcopenia has been identified as a poor prognostic factor [15], though the strength of its association with clinical outcomes in advanced melanoma remains controversial [16].

In an attempt to further investigate the impact of the above-mentioned modifiable factors on therapeutic responses in melanoma patients, we profiled the GM and fecal metabolome, and we thoroughly characterized the nutritional status (including anthropometry, body composition, and biochemical parameters) of patients with advanced melanoma prior to receiving targeted therapy or immunotherapy. To our knowledge, although exploratory, this paper represents the first study to consider such factors together in order to identify potential early integrated signatures of response to therapy in these patients.

## 2. Results

### 2.1. Demographic, Anthropometric, Body Composition, Physical Activity, Dietary, and Clinical Characteristics of the Study Population

Thirty-one patients with advanced melanoma who were candidates for first-line anti-PD-1 immunotherapy or targeted therapy were enrolled. Patients were stratified into the following groups according to their therapeutic response: (i) responders, i.e., those with a complete or partial response to therapy or with stable disease (≥6 months); and (ii) non-responders, i.e., those with progressive disease. Baseline characteristics for the entire cohort and for comparison between responders and non-responders are shown in Table 1 (demographic and clinical) and Table 2 (anthropometry, body composition, physical activity, and diet). Interestingly, all patients who were subsequently classified as responders were characterized by higher weight, body mass index (BMI), and total (TAT), visceral (VAT), subcutaneous (SAT), and intramuscular (IMAT) adipose tissue (*p* ≤ 0.05). They also had a lower neutrophil-to-lymphocyte ratio (NLR) than non-responders and were predominantly non-sarcopenic (*p* ≤ 0.02). Regarding the occurrence of adverse events during therapy, none of the patients experienced mucositis or other grade 3–4 adverse events, suggesting that there was a lack of correlation with therapeutic response.

Flow cytometric analysis of absolute cell counts for innate and adaptive immune subsets was performed in 10 responders and 4 non-responders. Despite the small sample size, responders had significantly lower counts of neutrophils (3641 vs. 5258) and monocytic myeloid-derived suppressor cells (MDSCs) (118 vs. 272) than non-responders (*p* = 0.04, Student’s *t* test).

### 2.2. Gut Microbiota Profiling

**Figure 1:**
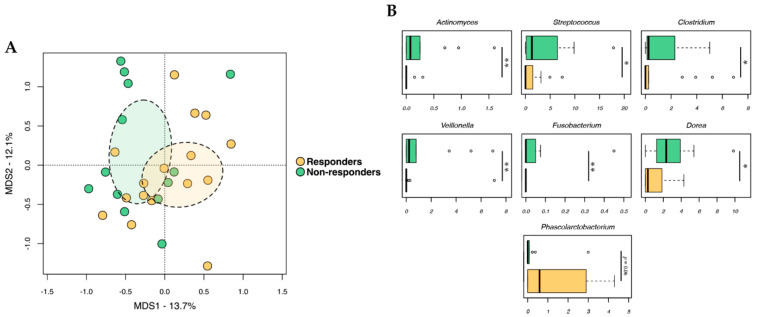
Baseline gut microbiota in advanced melanoma patients in relation to therapeutic responses. (**A**) PCoA based on the Jaccard similarity index between the gut microbiota profiles of responders and non-responders. Ellipses include a 95% confidence area based on the standard error of the weighted average of sample coordinates. Significant separation between groups was found (*p* = 0.034, Adonis). (**B**) Boxplots showing the relative abundance distribution of genera differentially represented between groups. Wilcoxon test, * for *p* < 0.05, ** for *p* < 0.01. For *Phascolarctobacterium*, only a non-significant trend of *p* = 0.06 was found.

To establish associations between treatment response and baseline GM profile, we compared alpha (i.e., intra-individual) and beta (i.e., inter-individual) diversity, as well as the compositional structure of GM between responders and non-responders. The pCoA plot of beta diversity based on the Jaccard similarity index revealed significant segregation between responders and non-responders (*p* = 0.034, Adonis) (Figure 1A). In contrast, no differences between groups were observed for alpha diversity (*p* ≥ 0.37, Wilcoxon test) From a taxonomic standpoint, responders were discriminated through an under-representation of *Actinomyces*, *Streptococcus*, *Clostridium*, *Veillonella*, *Fusobacterium*, and *Dorea* (*p* < 0.05) (Figure 1B). On the other hand, they tended to be enriched in *Phascolarctobacterium* (*p* = 0.06). Please review the Supplementary Figure S1 for phylum-level composition and differential representation of families between responders and non-responders.

### 2.3. Fecal Metabolomic Profile

**Figure 2:**
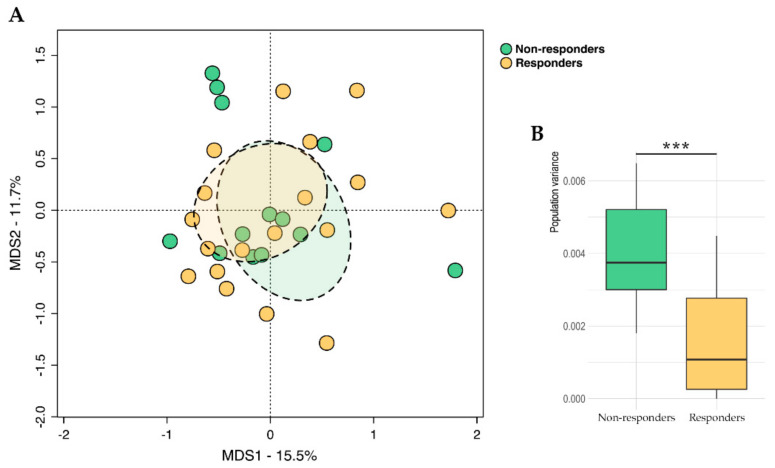
Fecal metabolome diversity at baseline in advanced melanoma patients in relation to therapeutic response. (**A**) PCoA based on the Euclidean distances between the fecal metabolomic profiles of responders and non-responders. Ellipses include 95% confidence areas based on the standard error of the weighted average of the sample coordinates. Significant separation between groups was found (*p* = 0.05, Adonis). (**B**) Boxplots showing the within-group variance in Euclidean distances. Wilcoxon test, *** for *p* < 0.001.

In parallel, responders and non-responders were compared in terms of their baseline fecal metabolomic profiles. Similar to the GM data, PCoA of beta diversity based on Euclidean distances revealed significant segregation based on therapeutic response (*p* = 0.05, Adonis) (Figure 2A), with non-responders also showing significantly higher within-group variance (*p* < 0.001, Wilcoxon test) (Figure 2B). The discriminating metabolites between groups are reported in Table 3 (*p* ≤ 0.05). Notably, butyric acid and its derivatives (i.e., methyl and propyl esters) were significantly over-represented in the non-responders (*p* ≤ 0.02).

### 2.4. Integration of Omics (Microbiomics and Metabolomics) Data and Patient Metadata

**Figure 3:**
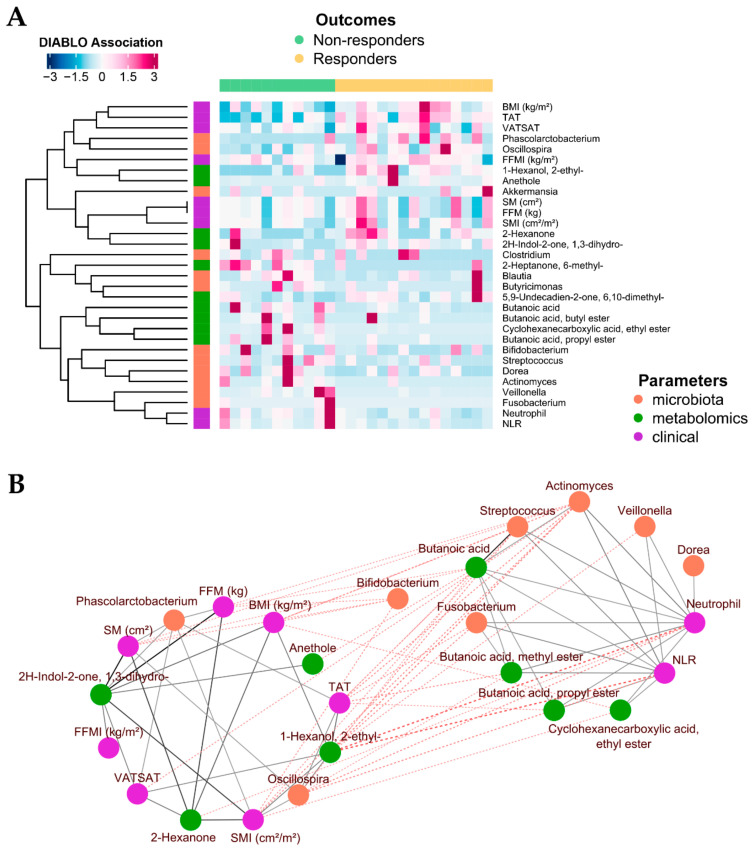
Integration of omics (microbiomics and metabolomics) data and host metadata in responder and non-responder patients with advanced melanoma. (**A**) Heatmap that shows associations between therapeutic response and bacterial genera, metabolites, body composition, and complete blood count parameters in advanced melanoma patients at baseline (i.e., before initiation of therapy). The plotted association values, which result from DIABLO, were scaled and trimmed to three standard deviation ranges. The contributions of all components generated via sPLS-DA were taken into account. (**B**) Association network between bacterial genera, metabolites, and host metadata. In brief, a pairwise similarity matrix was obtained from the latent components of sPLS by calculating the sum of the correlations between the original variables and each of the latent components of the model. Pairwise similarity values represent both positive and negative connections between all parameters in the different model blocks. Similarity connections are considered as edges that connect nodes in the network graph. Solid gray lines represent positive associations, while dashed red lines represent negative associations. The thickness of the line indicates the strength of the association. Two modules—shown as separate circles—were identified, and they contained features that were strongly associated with each other. BMI, body mass index; FFM, fat-free mass; NLR, neutrophil-to-lymphocyte ratio; SM, skeletal muscle; SMI, skeletal muscle index; TAT, total adipose tissue; VATSAT, visceral-to-subcutaneous adipose tissue ratio.

**Figure 4:**
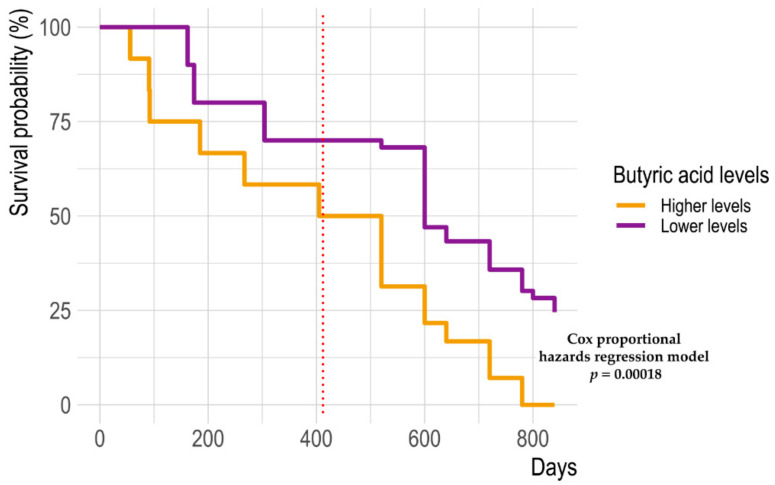
Elevated fecal levels of butyric acid at baseline are associated with a decreased survival rate in advanced melanoma patients. Kaplan–Meier curves for overall survival in the entire cohort. Patients were stratified based on basal fecal butyrate levels above or below the whole cohort median. Measurements were available through day 405 (vertical dashed red line), after which point a linear model trained on known data was used to predict future survival trends (through day 840). Cox proportional hazards model for days 0 to 405, *p* = 0.05; days 0 to 840, *p* = 0.00018.

Next, we aimed to find multivariate associations among GM profiles, metabolomic data, and clinical (i.e., NLR) and body composition datasets in relation to therapeutic response. To this end, we implemented an N-integration framework using multiblock sPLS-DA (see Materials and Methods for further details). By confirming the above results, we found that all datasets had discriminative features for classifying samples into non-responder and responder groups (Supplementary Figure S2). The magnitude of the associations between these parameters and therapeutic responses was then investigated using DIABLO, which largely confirmed and partially extended the previous results (Figure 3A). In particular, body composition parameters, such as BMI, TAT, the visceral-to-subcutaneous adipose tissue (VATSAT) ratio, skeletal muscle (SM), skeletal muscle index (SMI), and fat-free mass (FFM), were strongly associated with the responder group. On the other hand, non-responders showed strong associations with neutrophil count and NLR. In terms of microbial taxa, the therapeutic response was associated with *Oscillospira* and *Phascolarctobacterium*, while non-response was confirmed to be associated with *Fusobacterium*, *Veillonella*, *Streptococcus*, *Dorea*, *Clostridium*, and *Actinomyces*. From a metabolomic standpoint, higher levels of butyric acid and derivatives (i.e., butyl and propyl esters), along with higher levels of cyclohexanecarboxylic acid ethyl ester and 2-heptanone 6-methyl-, were strongly associated with non-responders. Finally, the therapeutic response was confirmed to be associated with 1-hexanol 2-ethyl-, 2H-indol-2-one 1,3-dihydro-, 2-hexanone, 5,9-undecadien-2-one 6,10-dimethyl-, and anethole. When reconstructing the network of associations derived via the integration analysis (Figure 3B), we identified the following distinct modules: (i) a response-associated module that linked the above-mentioned body composition parameters to *Phascolarctobacterium* and *Oscillospira*, as well as to the metabolites 2-hexanone, anethole, 2H-indol-2-one 1,3-dihydro-, and 1-hexanol 2-ethyl-; and (ii) a non-response-associated module that linked *Streptococcus*, *Actinomyces*, *Veillonella*, *Dorea*, and *Fusobacterium* to butyric acid (and derivatives) and cyclohexanecarboxylic acid ethyl ester, as well as higher levels of neutrophils (and NLR). Interestingly, higher basal fecal levels of butyric acid were also associated with decreased survival (Cox proportional hazards model, *p* ≤ 0.05) (Figure 4).

**Figure 5:**
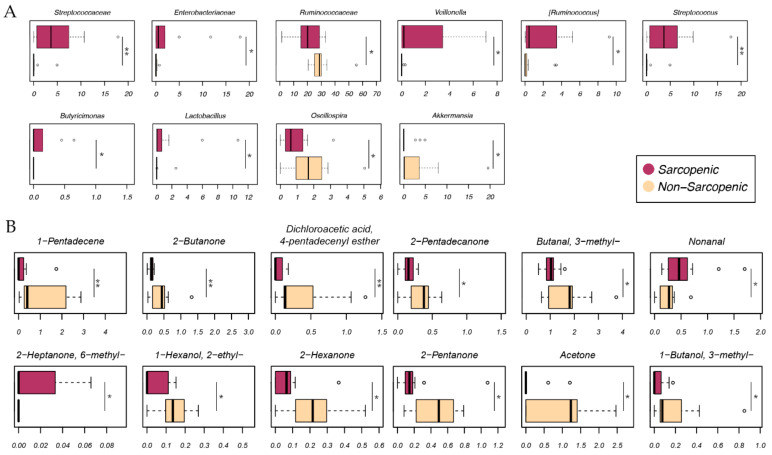
Gut microbiota and metabolome differences at baseline in advanced melanoma patients in relation to the presence of sarcopenia. Boxplots showing the distribution of the relative abundance of taxa (**A**) and the relative concentration (µg/g of internal standard) of metabolites (**B**) differentially represented between sarcopenic and non-sarcopenic patients. Wilcoxon test, * for *p* ≤ 0.05, ** for *p* < 0.01.

Finally, given that therapeutic response was inversely associated with the presence of sarcopenia, we specifically examined the differences in gut microbiota and metabolome between sarcopenic and non-sarcopenic patients (Figure 5). The gut microbiota of sarcopenic patients were enriched in *Streptococcaceae*, *Enterobacteriaceae*, *Veillonella*, *Ruminococcus*, *Streptococcus*, *Butyricimonas*, and *Lactobacillus*, while they were depleted in *Ruminococcaceae*, *Oscillospira*, and *Akkermansia* (*p* ≤ 0.05, Wilcoxon test). In addition, sarcopenic and non-sarcopenic patients differed in many metabolites, including 2-heptanone, 6-methyl-, and nonanal, which were overabundant in the former patient type, and 1-pentadecene, 2-butanone, dichloroacetic acid 4-pentadecenyl ester, 1-hexanol 2-ethyl-, 2-hexanone, 2-pentanone, acetone, 1-butanol 3-methyl-, 2-pentadecanone, and butanal 3-methyl-, which were overabundant in the latter patient type.

## 3. Discussion

**Figure 6:**
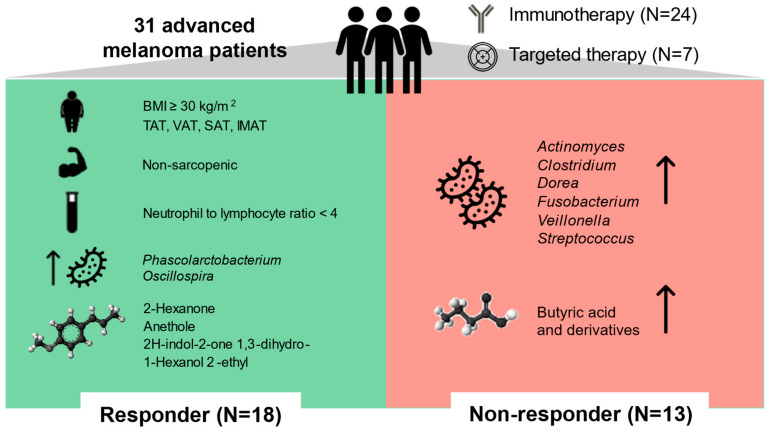
Summary of the study’s key findings. Thirty-one patients with unresectable IIIC-IV-stage cutaneous melanoma were characterized prior to initiation of targeted or first-line immunotherapy, anthropometry, body composition, nutritional status, physical activity, biochemical parameters, immunoprofiling, and fecal microbiome and metabolome. Patients subsequently classified as responders were found to be obese (with high body mass index (BMI) and high levels of total adipose tissue (TAT), visceral adipose tissue (VAT), subcutaneous adipose tissue (SAT), and intramuscular adipose tissue (IMAT)), non-sarcopenic, and enriched in certain fecal taxa (e.g., *Phascolarctobacterium*) and metabolites (e.g., anethole), which were potentially endowed with immunostimulatory and oncoprotective activities. They also had a lower neutrophil-to-lymphocyte ratio, which is an inflammatory biomarker of poor prognosis. On the other hand, non-response was associated with increased proportions of *Streptococcus*, *Actinomyces*, *Veillonella*, *Dorea*, *Fusobacterium*, higher neutrophil levels (and neutrophil-to-lymphocyte ratios), and higher fecal levels of butyric acid and its derivatives, which also correlated with decreased survival.

In this study, we identified an integrated set of GM, metabolomic and body composition features associated with therapeutic response in patients with advanced melanoma (Figure 6). Interestingly, these signatures were independent of the immunotherapy or targeted therapy received, and they likely represented common prognostic biomarkers. In particular, all patients subsequently classified as responders were obese, non-sarcopenic, and enriched in certain fecal taxa and metabolites prior to initiation of therapy. They also had higher levels of adipose tissue compared to non-responders and, as expected, lower levels of NLR, which is an inflammatory biomarker of poor prognosis [17], as well as lower counts of neutrophils and MDSCs, which are immune cell subsets associated with melanoma progression [18,19]. In particular, both MDSCs and tumor-infiltrating neutrophils are thought to exert immunosuppressive activity, as well as promote angiogenesis and tumor growth, thus contributing to immunotherapy resistance [20,21].

Although seemingly paradoxical, as discussed above, the relationship with obesity is not entirely surprising, as BMI has recently been found to be associated with better outcomes (namely, progression-free survival and overall survival) in patients with metastatic melanoma treated with targeted therapy or immunotherapy, which typically do not induce weight loss. The survival advantage in obese patients may be explained based on reverse causality, wherein patients with more aggressive disease have previous weight loss and BMI reduction, as well as enhanced “metabolic reserve” to withstand the wasting effects of cancer or its associated treatments [22]. Our study confirms, but also extends, this evidence by showing that therapeutic response is also associated with body composition parameters, such as TAT and VATSAT. Other authors have highlighted the potential link between SAT and sensitivity to PD-1/PD-L1 inhibition [23,24]. Furthermore, it should be remembered that SAT is the compartment responsible for leptin production [25]. As discussed by Wang et al. [26], obese patients would have an overexpression of PD-1, which is mainly expressed by T cells, due to the high leptin levels typical of obesity. Excess PD-1 would make T cells highly responsive to PD-1 inhibitors, paradoxically enhancing cancer responsiveness after anti-PD-1 therapy. However, it remains unclear why the association with obesity is not found in the chemotherapy setting, as well as how obesity-induced low-grade inflammation and immunosuppression could be an advantage [26,27]. Even the data on sarcopenia, although recently debated [16], are partly expected, as loss of skeletal muscle mass and function is strongly associated with poor outcomes and adverse events in several oncological contexts [28]. However, the mechanisms underlying the association between sarcopenia and poor response to anticancer therapy remain to be elucidated. In a clinical trial of advanced melanoma patients treated with anti-PD1 checkpoint inhibitors, Heidelberger et al. [29] observed that sarcopenic female patients had 6.5-fold higher anti-PD-1-related early acute limiting toxicity and no improvement in anti-tumor response. The authors hypothesized that this outcome may be related to weight-based dosing, which indicates high-dose drug administration, as it is assumed that pharmacokinetic parameters are altered in patients with high BMI, while drug distribution may be impaired due to loss of lean mass in sarcopenic patients. As for GM, responders were discriminated based on the presence of high proportions of *Phascolarctobacterium* and *Oscillospira*, as well as low proportions of *Actinomyces*, *Streptococcus*, *Fusobacterium*, *Clostridium*, *Veillonella*, and *Dorea*. Interestingly, *Phascolarctobacterium* was recently listed among the consistent taxonomic biomarkers associated with responsiveness to melanoma immunotherapy [30]. It should be mentioned that *Phascolarctobacterium* was also part of the 11-strain commensal consortium that was shown to robustly elicit interferon gamma-producing CD8 T cells in the intestine and improve the therapeutic efficacy of immunotherapy in tumor models [31]. The association between the opportunistic pathogens *Fusobacterium* and *Streptococcus* and poor prognosis (i.e., failure to respond to therapy) also confirms the validity of the available literature [9,14,32]. On the other hand, conflicting data are reported in the literature for *Veillonella*. Indeed, increased relative abundance of the family *Veillonellaceae* has been suggested to be an immunotherapy-favorable feature, though non-responder melanoma patients have been found to be enriched in *Veillonella atypica* [10]. These observations suggest the importance of high-resolution taxonomic profiling (down to the species level) in drawing reliable conclusions. With respect to metabolites, the most intriguing finding is arguably the over-representation of the short-chain fatty acid butyric acid and its derivatives (i.e., methyl and propyl esters) in the feces of non-responders. In addition to being present in some foods, particularly those containing bovine milk fat [33], butyrate is universally recognized as a microbiota metabolite (resulting from fiber fermentation) that is crucial for whole-body health [34]. Similarly, its derivatives are typically produced during polysaccharide fermentation processes and are generally recognized to have anti-inflammatory effects [35,36]. However, butyrate (and propionate) has recently been shown to limit the efficacy of immunotherapy (anti-CTLA-4). The mechanism behind this phenomenon is probably related to its immunomodulatory activity, i.e., induction of Treg cells, reduced accumulation of memory T cells, and lower inducible T cell co-stimulator induction in T cells [37]. Consistently, in our study, higher basal fecal levels of butyric acid were associated with decreased survival. Regarding the biochemical basis of high butyrate (and derivatives) levels in non-responders, it should be noted that no significant differences in fiber intake were found between responders and non-responders, suggesting a limited contribution of diet. On the other hand, butyrate is also produced via amino acid metabolic pathways by some pathobionts, including *Fusobacterium* [38], which was found to be enriched in non-responders. Finally, it was recently found that increasing butyric acid concentrations were associated with the relative abundance of several taxa, such as *Actinomyces*, *Streptococcus*, and *Veillonella* [39], which were also enriched in non-responders. On the other hand, responders showed elevated fecal levels of 2-hexanone, anethole, 2H-indol-2-one, 1,3-dihydro-, and 1-hexanol 2-ethyl. It should be noted that it is practically impossible to determine the origin of these metabolites, as they could originate from the diet or other environmental exposures, the host, the GM, or the metabolism (even combined) of the latter subject. For example, the presence of methyl ketone 2-hexanone in feces could be the result of ingestion of contaminated food/water, a waste product of industrial activities [40,41], or produced by gut microbes [42,43]. It should also be noted that the few studies that measured this metabolite in body fluids estimated low recovery in stool [40,41], which could lead us to speculate about impaired intestinal absorption in responders. Similarly, 1-hexanol 2-ethyl is a primary alcohol that occurs naturally in plants, though it is also contributed by micro-organisms [44,45]. Regarding anethole, although we were unable to distinguish its isoforms, it is worth noting that trans-anethole (from *Foeniculum vulgare* extracts) inhibited UV-induced melanogenesis in melanoma cells [46], thus suggesting an overall protective role. Anethole has also been shown to trigger apoptosis, autophagy, and oxidative stress in several cancer cells [47,48], further supporting its association with better prognosis.

The following main limitations of the study should be mentioned: (i) the small sample size, mainly due to the COVID-19 pandemic, and, related to this issue, the impossibility of collecting whole blood samples and performing the expected analyses in all subjects, thus limiting the relevance of our findings, especially those related to the immunological profile; (ii) the age range of the whole cohort, including older adults, whose GM, for example, may have already changed [49]; (iii) sarcopenia was defined based on the values of SMI, rather than by the recently updated algorithm published by the European Working Group on Sarcopenia in Older People [50]; (iv) the single-timepoint analysis, which did not allow us to evaluate the changes over time in the measured variables; and (v) the associative nature of the study.

## 4. Materials and Methods

### 4.1. Patient Enrollment and Sample Collection

Consecutive patients with unresectable IIIC-IV-stage cutaneous melanoma were prospectively evaluated at the Fondazione IRCCS Istituto Nazionale dei Tumori in Milan, Italy, before they started first-line anti-PD-1 immunotherapy (nivolumab or pembrolizumab) or targeted therapy (dabrafenib and trametinib), between September 2019 and December 2020. Inclusion criteria were as follows: (i) age ≥ 18 years and ≤85 years; (ii) 0, 1, or 2 Performance Status assessment using the ECOG (Eastern Cooperative Oncology Group) Score, which assesses each patient’s level of functional status and ability to perform self-care; and (iii) at least one measurable lesion, as assessed via computed tomography (CT) or magnetic resonance imaging (MRI) per Response Evaluation Criteria in Solid Tumors version 1.1 (RECIST v1.1). Subjects with treated brain metastases without MRI evidence of progression with untreated brain metastases, who were neurologically asymptomatic without systemic corticosteroids for at least two weeks prior to cancer therapy, were also included. Exclusion criteria included a history of ocular/uveal melanoma; the presence of active brain metastases, leptomeningeal disease, autoimmune disease, type I diabetes mellitus, hypothyroidism requiring only hormone replacement, inflammatory bowel disease, celiac disease, or documented food allergy; and prior active cancer in the past three years, except for localized cancers that had been cured and did not recur.

All enrolled patients were characterized based on their nutritional status, physical activity, and biochemical and inflammatory measures, as described below. Blood and fecal samples were collected from each patient prior to the initiation of therapy. Blood samples were used for complete blood count, including the NLR and immune profile. NLR is an inflammatory biomarker of clinical interest in the prognosis of solid tumors, especially melanoma [17], with values ≥ 4 being associated with decreased overall survival [51]. Fecal samples were immediately stored at −80 °C and shipped on dry ice to the Department of Pharmacy and Biotechnology (University of Bologna, Bologna, Italy), where they were stored at −80 °C until being processed for microbiome and metabolome profiling.

At the end of treatment, efficacy was assessed, with the RECIST v1.1 criteria used as the reference standard. Based on their radiographic response, patients were classified as responders (for complete response, partial response, or stable disease for ≥6 months) or non-responders (for progressive disease).

The study protocol was approved by the Ethics Committee of the Fondazione IRCCS Istituto Nazionale dei Tumori in Milan (registration number 126/18, 19 July 2018). It was conducted according to the guidelines of the Declaration of Helsinki. Written informed consent was obtained from all patients who participated in the study.

### 4.2. Nutritional Status Assessment

The assessment of nutritional status included the collection of anthropometric data (weight and height) for the calculation of the BMI in kg/m^2^, as well as the detection of sarcopenia based on the body composition assessment. BMI values were stratified according to the World Health Organization’s cut-off points [52]. Body composition was assessed via CT. CT scans performed for diagnostic purposes (≤30 days before the first day of cancer therapy) were used to measure muscle mass, and the third lumbar vertebra (L3) was selected as the standard landmark. Extrapolated L3 images were segmented using Slice-O-Matic v4.3 software (Tomovision, Montreal, QC, Canada) to measure areas (cm^2^) of SM and TAT, which were defined as the sum of SAT, IMAT, and VAT. The skeletal muscle index (SMI) was calculated by dividing SM (kg) by the square of height (m^2^). Sarcopenia was defined using published sex-specific cut-offs; these cut-offs were SMI < 38.5 and 52.4 cm^2^/m^2^ for females and males, respectively [53]. The regression equation of Mourtzakis et al. [54] (0.30 × SM + 6.06) was used to calculate whole-body fat-free mass (FFM) in kg.

### 4.3. Physical Activity and Dietary Questionnaires

Patients were administered the following questionnaires: (i) the International Physical Activity Questionnaire Short Form (IPAQ-SF) [55], which was used to estimate physical activity levels based on activity performed during the past 7 days; and (ii) the EPIC Food Frequency Questionnaire (FFQ), which is a validated semi-qualitative questionnaire used to assess dietary habits over the past year [56].

Based on the IPAQ, patients were categorized as inactive [<700 Metabolic Equivalent of Task-min/week (MET)], sufficiently active (700-2519 MET), or very active (>2520 MET). For the EPIC FFQ, patients were asked to answer 188 questions regarding the amount and frequency of food consumption using standard reference units and a photographic food atlas with portion sizes. For fiber intake, the cut-off value of 25 g/day was chosen because it is the amount recommended by the Dietary Reference Values for Italian (LARN) [57] and European (EFSA) [58] populations. Furthermore, adherence to the Mediterranean diet was determined using the Italian Mediterranean Index (IMI) [59], which defined the following 4 categories: minimal adherence (0–1), discreet adherence (2–3), good adherence (4–5), and maximal adherence (6–11).

### 4.4. Immune Profile

The immune profile was evaluated using a 10-color Gallios cytometer (Beckman Coulter, Brea, CA, USA). Due to the COVID-19 pandemic, whole blood was collected from only 14 of the 31 patients. In fact, during the pandemic, all hospitals in Italy decided to stop collecting fresh whole blood samples unless absolutely necessary to avoid the possible risk of infection of healthcare workers. All samples were collected in BD Vacutainer^®^ K2E (EDTA) (BD 367525) and stored at room temperature for less than 2 h to enable the counting of populations with short half-lives, such as neutrophils and eosinophils [60]. To determine absolute leukocyte counts from fresh peripheral whole blood samples, we used Trucount™ Absolute Counting Tubes (Becton Dickinson, Franklin Lakes, NJ, USA, 663028). In total, 100 µL of freshly isolated blood was stained using the recommended volume of the following specific antibody reagents: CD15-FITC (BD-555401), CD19-PE (BioLegend-302208), HLA-DR-PE-CF594 (BD-562208), CD16-PE-Cy7 (BD-557744), CD33-APC (BD-551378), CD45-APC-AI700 (Coulter-A79390), CD14-APC-Fire750 (BioLegend-367120), CD56-BV421 (BD-562751), and CD3-BV510 (BD-563109), as well as FcR Blocking Reagent (Miltenyi) to avoid non-specific signals, and incubated at 4 °C in the dark for 30 min. NH_4_Cl (ACK) was added to eliminate red blood cells and incubated for 10 min at room temperature, being kept in darkness throughout this stage. Finally, cells were acquired via flow cytometry, in which the optical alignment and fluidics of the instrument were routinely checked using Flow-Check Fluorospheres (Beckman Coulter, A63493), while Flow-Set Fluorospheres (Beckman Coulter, A63492) were used to control light scatter and fluorescence intensity. Data were analyzed using FlowJo software (v.10.8.0, FlowJo, Franklin Lakes, NJ, USA). The panel used allowed simultaneous quantification of 31 immune subsets, as previously described [61], including neutrophils (CD16+ CD15+), eosinophils (CD16− CD15+), T cells (CD3+) and their activation states (CD3+ HLA-DR+), B cells (CD19+ HLA-DR+), NK cells (CD3− CD56+) and their subsets (based on differential expression of CD16 and CD56), NKT-like cells (CD3+ CD56+), three monocyte subsets (differentiated by CD14 and CD16), and myeloid-derived suppressor cells (MDSCs) (via expression of HLA-DR-/low CD33+ and differential expression of CD14 and CD15).

### 4.5. Microbial DNA Extraction and 16S rRNA Amplicon Sequencing

Microbial DNA was extracted from a 0.25-gram aliquot of fecal sample from each of 31 patients, and this stage was carried out using the repeated bead-beating protocol [62] with only minor modifications [63]. In brief, stool samples were resuspended in 1 mL of lysis buffer in the presence of four 3-millimeter glass beads and 0.5 g of 0.1-millmeter zirconia beads (BioSpec Products, Bartlesville, OK, USA), before being bead-beaten three times at 5.5 movements/s for 1 min in a FastPrep homogenizer (MP Biomedicals, Irvine, CA, USA). After 15 min of incubation at 95 °C, supernatants were separated via centrifugation at 13,000 rpm for 5 min and incubated with 260 µL of 10-molarity ammonium acetate and one volume of isopropanol for 30 min. Nucleic acid pellets were washed with 70% ethanol and resuspended in 100 µL of TE buffer. RNA was removed via incubation using 2 µL of dNase-free rNase (10 mg/mL) at 37 °C for 15 min. The DNeasy Blood and Tissue Kit (QIAGEN, Hilden, Germany) was used to perform subsequent DNA purification steps. DNA concentration and quality were assessed using the NanoDrop ND-1000 spectrophotometer (NanoDrop Technologies, Wilmington, DE, USA).

The V3–V4 hypervariable regions of the 16S rRNA gene were amplified using the 341F and 785R primers with Illumina adapter overhang sequences, as previously described [63]. PCR products were purified using a magnetic bead-based system (Agencourt AMPure XP; Beckman Coulter), followed by sample indexing via limited-cycle PCR using Nextera technology. Indexed libraries were purified via a further clean-up step, as described above, and pooled at an equimolar concentration of 4 nM. Sequencing was performed by loading denatured and 5-picometer-diluted libraries onto an Illumina MiSeq platform using the 2 × 250 bp paired-end protocol based on the manufacturer’s instructions (Illumina, San Diego, CA, USA).

### 4.6. Fecal Metabolomics

A portion of the fecal samples (approximately 10 g) for each of the 31 patients was shipped on dry ice to the Department of Soil, Plant, and Food Science, the University of Bari Aldo Moro, Bari, Italy, for metabolomic analysis. The samples, which were placed in 10-millilter glass vials, were sealed with polytetrafluoroethylene-coated silicone rubber septa and equilibrated for 10 min at 40 °C. Upon completion of sample equilibration, a conditioned 50/30-µm DVB/CAR/PDMS fiber (Supelco, Bellefonte, PA, USA) was exposed to headspace for 40 min to extract volatile compounds using the CombiPAL system injector autosampler (CTC Analytics, Zwingen, Switzerland). Volatile organic compounds (VOCs) were thermally desorbed by immediately transferring the fiber into the heated injection port (220 °C) of a Clarus 680 (Perkin Elmer, Beaconsfield, UK) gas chromatograph equipped with a Rtx-WAX column (30-m × 0.25-mm i.d., 0.25-μm film thickness) (Restek, Cernusco sul Naviglio, Milan, Italy), which was coupled to a Clarus SQ8MS (Perkin Elmer) with source and transfer line temperatures maintained at 250 and 210 °C, respectively. Injection was performed in splitless mode using helium as the carrier gas at a flow rate of 1 mL/min. The oven temperature was initially set at 35 °C for 8 min, and it was then increased to 60 °C at 4 °C/min, to 160 °C at 6 °C/min, and, finally, to 200 °C at 20 °C/min and held for 15 min. Electron ionization masses were recorded at 70 eV in the 34–350 mass-to-charge ratio interval. Each chromatogram was analyzed for peak identification using the National Institute of Standard and Technology 2008 (NIST) library. A peak area threshold of >1,000,000 and a match probability of 85% or greater were used for VOC identification, followed by manual visual inspection of the fragment patterns when necessary. 4-Methyl-2-pentanol (final concentration, 33 mg/L) was used as the internal standard (IS) in all analyses to quantify the compounds via interpolation of the relative areas in comparison to the IS area (expressed as μg/g of IS).

### 4.7. Bioinformatic and Statistical Analysis

Raw sequences were processed using a pipeline that combined PANDAseq [64] and QIIME 2 [65]. Specifically, paired-end sequences were first assembled into a single-end amplicon using PANDAseq, retaining only assembled reads in the range of 350 to 500 nucleotides. An error correction step was then performed using the USEARCH11 ‘fastq_filter’ module [66], which set a maximum error rate of 0.03 to discard low-quality sequences. Sequences were then binned into amplicon sequence variants (ASVs) using the DADA2 pipeline [67], with chimeras being removed at the same time. Taxonomic assignment was performed using the VSEARCH algorithm [68] to align ASVs with the Greengenes database v13.8. Alpha diversity was computed using the ‘diversity core-metrics-phylogenetic’ plugin from QIIME 2, as well as multiple metrics, including the number of observed ASVs, the Shannon index, and Faith’s Phylogenetic Diversity. Beta diversity was estimated via the same plugin using both quantitative and qualitative metrics, such as weighted/unweighted UniFrac, Jaccard, and Euclidean measures, which were used for principal coordinates analysis (pCoA).

All statistical analyses were performed using R/rStudio 4.2.2 software. The pwr R package (https://CRAN.R-project.org/package=pwr, accessed on 2 June 2022) was implemented to perform a post hoc power analysis. For this procedure, the alpha value was set at 0.05, and the effect size was estimated based on the response rate. The resulting power was 0.88, which could be considered sufficiently robust given the exploratory nature of this study. pCoA plots were generated using the vegan R package v2.6-4 (http://www.cran.r-project.org/package=vegan/, accessed on 2 June 2022), and data separation was tested via permutational analysis of variance with pseudo-F ratios (Adonis tests). The variance in beta diversity of a given group, i.e., the distance in beta diversity between samples within the same group, was calculated as the average squared distance to the mean. The Wilcoxon test with continuity correction was used to compare alpha diversity, compositional structure, and metabolite levels between groups. To find multivariate associations among GM profiles, metabolomic data, and patient metadata, we employed multi-block sparse partial least square discriminant analysis (sPLS-DA), as implemented in the DIABLO/mixOmics packages in R [69]. The model developed for this approach was tuned according to the developer’s guidelines (http://mixomics.org, accessed on 30 June 2022), with repeated cross-validation performed with 10 folds and 100 repeats. The approach allowed us to pick the best number of components for sPLS-DA computation, reducing the overall bit error rate. A similar tuning approach was then implemented, based on the developer’s guidance, to select the appropriate features retained from the different layers of information for the DIABLO model. Results were first visualized via plotting the individual values for each data layer (plotIndiv function of the mixOmics package), which represented the contribution of each layer to patient stratification. Next, clustered image maps were generated using cimDiablo to highlight the relationships between the multi-omics panel and the outcome variable. Finally, the network function of mixOmics was used to construct a relevance network that consisted of a set of features linked via a DIABLO association value, which we considered to be a network edge list and plotted as a network plot using Cytoscape [70]. In brief, a pairwise similarity matrix was obtained from the latent components of sPLS by calculating the sum of the correlations between the original variables and each of the latent components of the model. Pairwise similarity values represented both positive and negative connections between all parameters in the different model blocks (i.e., GM genera, metabolites, and host metadata). Similarity connections were considered to be edges that connected nodes in a network graph, thus enabling the detection of network modules (using the cluster_spinglass algorithm from the igraph package). Patients were stratified based on basal fecal butyrate levels above or below the whole cohort median, and survival predictions were made using a linear model trained using survival information collected from the start of therapy until 405 days later, inferring the likelihood of survival for at least 840 days. For both known survival and overall survival, including predicted values, we tested the significance of the separation between groups with a Cox proportional hazard model, which employed the “Surv” and “coxph” functions of the survival R package v3.5-0 (https://www.cran.r-project.org/package=survival/, accessed on 30 June 2022). For demographic, anthropometric, body composition, physical activity, dietary, and clinical characteristics of the patients, data were summarized as either means and standard deviations (SD) or absolute frequencies and percentages (%). Statistical comparisons for categorical and numerical variables were performed using Fisher’s test, Wilcoxon test, or Student’s *t*-test, as appropriate. A *p* value ≤ 0.05 was considered to be statistically significant; a *p* value ≤ 0.1 was considered to represent a trend.

## 5. Conclusions

By integrating omics (microbiomics and metabolomics) data and host metadata (in particular body composition), our study, while exploratory, provided a list of potential early biomarkers of response to therapy in patients with advanced melanoma. In particular, our findings highlight the relevance of obesity, lack of sarcopenia, and enrichment of microbial taxa endowed with immunostimulatory activities and oncoprotective metabolites as favorable prognostic signatures. Further studies in larger cohorts, possibly using longitudinal sampling, are needed not only to validate and deepen the GM findings, but also to further investigate the seemingly paradoxical relationship between obesity and therapeutic response in patients with melanoma (as well as other types of cancer). To this end, animal models should be used to move beyond the associations and unravel the underlying mechanisms. Once acquired, this body of knowledge will improve the clinical management of patients with advanced melanoma, especially the design of appropriate adjuvant therapeutic strategies to improve treatment response and support long-term health improvements. In the future, mathematical models that take into account a variety of host variables will be required to identify effective personalized treatment protocols.

## Figures and Tables

**Table 1:** Demographic and clinical characteristics of patients with advanced melanoma. Data are presented for the entire cohort and for responders and non-responders. Differences between groups were evaluated via Fisher’s test, Wilcoxon test, and Student’s *t*-test, as appropriate. *p* values ≤ 0.05 are shown in bold.

Characteristic	Overall (n = 31)	Responders (n = 18)	Non-Responders (n = 13)	*p* Value
Age (years), mean (SD)	62 (11)	62 (11)	61 (12)	0.5748
Sex, n (%)				0.4120
Male	23 (74)	12 (52)	11(48)	
Female	8 (26)	6 (75)	2 (25)	
Stage, n (%)				0.6207
IIIC	4 (13)	3 (75)	1 (25)	
IV	27 (87)	15(56)	12 (44)	
ECOG performance status, n (%)				0.1337
0	26 (84)	17 (65)	9 (35)	
1–2	5 (16)	1 (20)	4 (80)	
Planned anti-PD-1 treatment, n (%)				0.7777
Nivolumab	19 (61)	12 (63)	7 (37)	
Pembrolizumab	5 (16)	3 (60)	2 (40)	
Targeted therapy, n (%)				0.4130
Dabrafenib and trametinib	7 (23)	3 (43)	4 (57)	
NLR ^a^, mean (SD)	5.5 (7.5)	2.8 (1.6)	9.0 (10.4)	**0.0034**
NLR ^a^				**0.0196**
<4, n (%)	21 (70)	15 (71)	6 (29)	
≥4, n (%)	9 (30)	2 (22)	7 (78)	
Medication use ^b^, n (%)				
Antibiotics ^a^	10 (33)	5 (50)	5 (50)	0.4611
Probiotics ^a^	6 (20)	3 (50)	3 (50)	0.6599
Proton-pump inhibitors	7 (23)	2 (29)	5 (71)	0.0994
Corticosteroids	6 (19)	2 (33)	4 (66)	0.2076

ECOG, Eastern Cooperative Oncology Group; NLR, neutrophil-to-lymphocyte ratio; SD, standard deviation. ^a^ Data are missing for one patient. ^b^ During the past six months.

**Table 2:** Anthropometry, body composition, physical activity, and dietary characteristics of patients with advanced melanoma. Data are presented for the entire cohort and both responders and non-responders. Differences between groups were evaluated via either Fisher’s test, Wilcoxon test, or Student’s *t*-test, as appropriate. *p* values of ≤0.05 are shown in bold.

Characteristic	Overall (n = 31)	Responders (n = 18)	Non-Responders (n = 13)	*p* Value
Anthropometry				
Height (m), mean (SD)	1.71 (0.1)	1.72 (0.1)	1.71 (0.1)	0.6591
Weight (kg), mean (SD)	77.9 (17.4)	84 (18.1)	69.5 (12.5)	**0.0291**
BMI (kg/m^2^), mean (SD) BMI, n (%)	26.5 (4.8)	28.5 (4.9)	23.8 (3.2)	**0.0073** **0.0275**
<30 kg/m^2^	25 (81)	12 (48)	13 (52)	
≥30 kg/m^2^	6 (19)	6 (100)	0 (0)	
CT ^a^				
Sarcopenic	14 (56)	6 (43)	8 (57)	**0.0029**
Non-sarcopenic	11 (44)	11 (100)	0 (0)	
SM (cm^2^/m^2^), mean (SD)	135.2 (29.4)	139.5 (32.5)	126.0 (20.0)	0.3983
TAT (cm^2^/m^2^), mean (SD)	125.21 (118.63)	149.18 (131.2)	74.28 (62.1)	**0.0018**
VAT (cm^2^/m^2^), mean (SD)	172.2 (123.4)	207.8 (131.1)	96.7 (57.7)	**0.0113**
SAT (cm^2^/m^2^), mean (SD)	191.0 (89.4)	225.3 (86.2)	118.1 (38.7)	**0.0010**
IMAT (cm^2^/m^2^), mean (SD)	12.4 (10.1)	14.5 (10.7)	8.0 (7.7)	**0.0510**
VATSAT, mean (SD)	0.88 (0.45)	0.93 (0.47)	0.77 (0.41)	0.3983
FFM (kg), mean (SD)	46.6 (8.8)	47.9 (9.8)	43.0 (5.9)	0.4025
Physical activity, n (%) ^b^				0.1516
Low	14 (45)	6 (43)	8 (57)	
Moderate	11 (36)	9 (82)	2 (18)	
High	6 (19)	3 (50)	3 (50)	
Fiber: EPIC FFQ ^c^				1.000
<25 g/die	17 (65)	12 (71)	5 (29)	
≥25 g/die	9 (35)	6 (67)	3 (33)	
Italian Mediterranean Index ^c^				0.0838
0–3	11 (42)	10 (91)	1 (9)	
4–11	15 (58)	8 (53)	7 (47)	

BMI, body mass index; CT, computed tomography; FFM, fat-free mass; FFQ, food frequency questionnaire; IMAT, intramuscular adipose tissue; SAT, subcutaneous adipose tissue; SD, standard deviation; SM, skeletal muscle; TAT, total adipose tissue; VAT, visceral adipose tissue; VATSAT, the ratio of visceral to subcutaneous adipose tissue. ^a^ Data are missing for six patients. ^b^ Assessed using the International Physical Activity Questionnaire. ^c^ Data are missing for five patients.

**Table 3:** Fecal metabolites differentially represented at baseline in advanced melanoma patients in relation to therapeutic response. For each metabolite, the mean concentration (µg/g of internal standard) values (±the standard error of the mean) in responders and non-responders, as well as the *p* value of the comparison (determined via Wilcoxon test), are reported.

Metabolite	Non-Responders	Responders	*p* Value
Butanoic acid, methyl ester	0.47 ± 0.1	0.013 ± 0.0063	0.002
1-Hexanol, 2-ethyl-	0.034 ± 0.01	0.15 ± 0.015	0.003
2-Heptanone, 6-methyl-	0.031 ± 0.0045	0.0069 ± 0.0025	0.006
Cyclohexanecarboxylic acid, ethyl ester	0.41 ± 0.12	0.0082 ± 0.0029	0.007
Butanoic acid	25 ± 4.3	5.7 ± 0.63	0.02
Butanoic acid, propyl ester	1.3 ± 0.36	0.1 ± 0.035	0.02
2H-Indol-2-one, 1,3-dihydro-	0.11 ± 0.037	0.12 ± 0.011	0.02
2-Hexanone	0.076 ± 0.022	0.18 ± 0.021	0.04
5,9-Undecadien-2-one, 6,10-dimethyl-	0.13 ± 0.024	0.34 ± 0.047	0.04
Anethole	0.14 ± 0.05	0.86 ± 0.26	0.05
Propanoic acid, ethyl ester	0.12 ± 0.037	0.007 ± 0.0035	0.05

## Data Availability

Sequencing reads were deposited in the National Center for Biotechnology Information’s Sequence Read Archive (NCBI SRA; BioProject ID PRJNA991471).

## Supplementary Materials

The following supporting information can be downloaded via the following link: https://www.mdpi.com/article/10.3390/ijms241411611/s1.
