# Supplementary material for: Gut Microbiota, Metabolome, and Body Composition Signatures of Response to Therapy in Patients with Advanced Melanoma
Source: Int J Mol Sci. 2023 Jul 18;24(14):11611. doi: 10.3390/ijms241411611 (PMC10380337; doi:10.3390/ijms241411611)
Supplement: Supplementary file 1 [file ijms-24-11611-s001.zip › ijms-2440123-supplementary.pdf]

Supplementary material

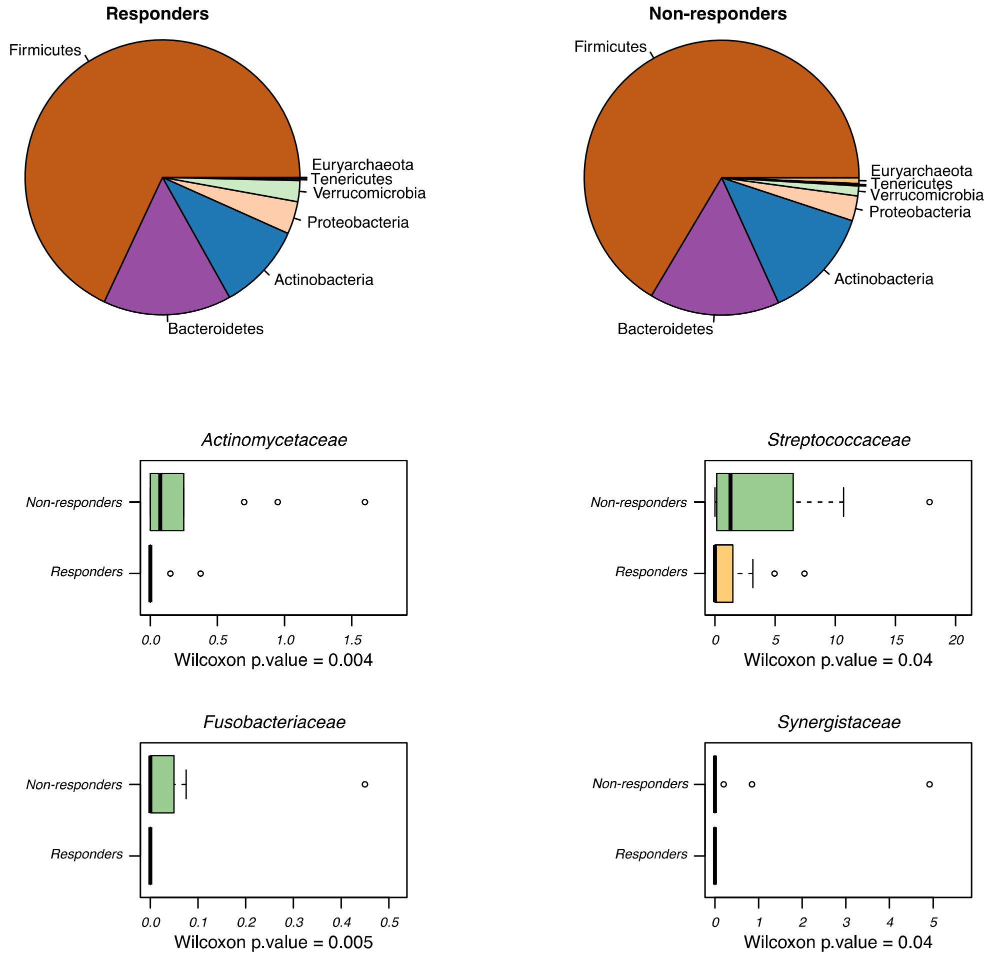

Figure S1. Baseline phylum- and family-level composition of the gut microbiota in advanced melanoma patients in relation to therapeutic response.

Top, Pie charts representing the phylum-level composition of the gut microbiota of responders and non-responders. Bottom, Boxplots showing the relative abundance distribution of families differentially represented between groups ($p \leq 0.05$, Wilcoxon test).

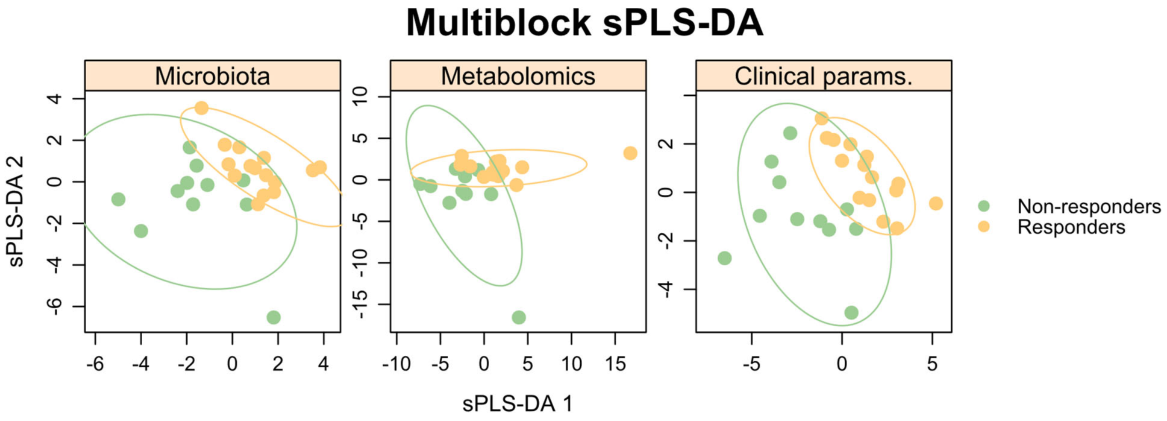

Figure S2. Sparse Partial Least Squares Discriminant Analysis of omics (microbiomics and metabolomics) data and patient metadata at baseline.

Multiblock analysis of baseline stratified data allowed to evaluate the contribution of each block to the distribution of samples in the non-responder and responder groups, scoring all of them as discriminant of the outcome. For clinical parameters, neutrophil to lymphocyte ratio and body composition data were used.
